# Agronomic evaluation of polymer-coated urea and urease and nitrification inhibitors for cotton production under drip-fertigation in a dry climate

**DOI:** 10.1038/s41598-020-57655-x

**Published:** 2020-01-30

**Authors:** Yanyan Li, Mingfang Hu, Mario Tenuta, Zhiwen Ma, Dongwei Gui, Xiangyi Li, Fanjiang Zeng, Xiaopeng Gao

**Affiliations:** 1 State Key Laboratory of Desert and Oasis Ecology, Xinjiang Institute of Ecology and Geography, Chinese Academy of Sciences, Urumqi, 830011 China; 2 Cele National Station of Observation and Research for Desert-Grassland Ecosystem, Cele, 848300 Xinjiang China; 3 University of Chinese Academy of Sciences, Beijing, 100049 China; 4 Department of Soil Science, University of Manitoba, Winnipeg, MB R3T 2N2 Canada

**Keywords:** Agroecology, Environmental impact

## Abstract

Interest in the use of enhanced-efficiency nitrogen (N) fertilizers (EENFs) has increased in recent years due to their potential to increase crop yield and reduce environmental N loss. Drip-fertigation is widely used for crop production in arid regions to improve water and nutrient use efficiency whereas the effectiveness of EENFs with drip irrigation remains unclear. A field experiment was conducted in 2015 and 2016 to examine the effects of EENFs on yield, N use and quality of cotton (*Gossypium hirsutum*) grown under drip-fertigation in arid NW China. Treatments included an unfertilized control and application of 240 kg N ha^−1^ by polymer-coated urea (ESN), urea alone, or urea plus urease (NBPT) and nitrification (DCD) inhibitors. ESN was all banded in the plant row at planting, whereas urea was applied with 20% N banded at planting and 80% N by six fertigation events over the growing season. Results showed there was generally no treatment effect on seed and lint yield, N concentration or allocations, N recovery efficiency and fiber quality index of cotton. A lack of treatment effect could be due to N supplied with drip-fertigation better synthesized with crop N needs and the relatively high soil native NO_3_^−^ availability, which hindered the effect of polymer-coated urea and double inhibitors. These results highlight the challenge of the employment of EENFs products for drip-fertigation system in arid area. Further research is required to define the field conditions under which the agronomic efficiency of EENFs products may be achieved in accordance with weather conditions.

## Introduction

Nitrogen (N) is often the most limiting nutrient in agricultural production systems. Large quantities of N fertilizers are usually applied to achieve maximum yields whereas fertilizer N use efficiency (NUE) are only 30 to 50% for crops grown on most agricultural soils^1^. Urea (46-0-0) is the most commonly used N fertilizer worldwide, due to its high N content, low cost, and ease of transport, storage and application^2^. However, urea is also identified as a low effective N source for crops because it can be lost through multiple pathways such as ammonia volatilization, nitrate leaching and N_2_O emission after being applied to soils^3^, which may cause risks to the environment and human health. Therefore, effective N fertilizer management practices are needed to improve NUE and crop yield while reducing its negative effect on environment.

Enhanced efficiency N fertilizers sources (EENFs) are developed to improve NUE and reduce N losses to environment. These include polymer-coated urea such as Environmentally Smart N (ESN, 44-0-0) and stabilized urea containing urease and/or nitrification inhibitors. Among the inhibitors, urease inhibitor N-(n-butyl) thiophosphorictriamide (NBPT) and nitrification inhibitor dicyandiamide (DCD) are widely used in agriculture systems to reduce the conversion from urea to NH_3_ plus ammonium (NH_4_^+^) and from NH_3_ plus NH_4_^+^ to NO_3_^−^, respectively. Previous studies have reported the effectiveness of ESN and urease and nitrification inhibitors to improve crop yields and reduce N losses to environment, whereas results were inconsistent. For example, use of NBPT with urea reduced NH_3_ volatilization loss and further improved N uptake and yield of flooded rice^4^. Use of DCD effectively increased yield and NUE of winter wheat^5^ and grassland pasture^6^. Other studies also showed positive response of crop yield and NUE with the use of ESN^7,8^. In contrast, Malhi *et al*. found that ESN did not increase barley yield, compared to conventional urea^9^. Watts *et al*. also reported ESN produced similar lint yield as urea whereas improved fiber quality for irrigated cotton grown in Coastal Plains of United States^10^. Use of ESN and SuperU which is a double-inhibitor product containing NBPT and DCD did not increase yield while reduced N_2_O emissions of irrigated potato in Manitoba of Canada^11^. In a recent meta-analysis, Li *et al*. summarized studies between 1980 and 2016 and found the effectiveness of EENFs were highly dependent on environmental conditions such as climate, soil and crops^12^. For example, the effectiveness of EENFs on yield and NUE were highly inhibited for crops grown in dry land systems due to limitation of water availability. Further site-specific studies are therefore required to examine the effectiveness of EENFs in various cropping systems.

Xinjiang province in NW China is an arid region with annual precipitation of only 161 mm on average. Developing water-saving irrigation strategy is of great importance in this region. In recent years, drip irrigation system has widely taken place of the traditional furrow irrigation due to its benefits on improving water and nutrient use efficiency. Cotton (*Gossypium hirsutum*) is the main cash crop in Xinjiang province with planting area accounting for 54% of total acreage of cotton in China^13^. Over 85% of cotton production in Xinjiang province is produced under drip irrigation system. In this system, water-soluble mineral fertilizers, commonly urea, are split-applied with irrigation water (fertigation) to better match crop N needs. A few studies have investigated the effect of urease and nitrification inhibitors on productivity and NUE of cotton under the drip-fertigated cropping system. For example, in the same area as in the current study, Liu *et al*. found that use of a nitrification inhibitor nitrpyrin did not affect yield but increased NUE of cotton under drip-fertigation system^14^. For irrigated corn in Spain, use of a new nitrification inhibitor DMPSA and drip-fertigation did not affect N uptake or yield but reduced N losses through N_2_O emissions, compared with the conventional system (sprinkler irrigation without inhibitors)^15^. It remains unknown whether double inhibitors of NBPT and DCD can slow N transformation to match crop N needs under drip-fertigation system. In addition, to our knowledge, no research has investigated the effectiveness of polymer-coated urea such as ESN under drip-fertigation system.

The objective of this study was to evaluate the effect of ESN and urease (NBPT) and nitrification (DCD) inhibitors on yield, N uptake and allocation, and fiber quality of cotton grown under drip-fertigation system. We hypothesized that the EENFs could better match crop N needs and thus improve productivity and fertilizer NUE.

## Results

### Overview of environmental conditions

Total precipitation over the growing season (April to October) was 88 mm in 2015 and 141 mm in 2016, respectively. The amount of irrigation was 450 mm in 2015 and 405 mm in 2016, amounting to 84% and 74% of total water input in the respective crop year. Air temperatures generally increased from April to July and decreased thereafter, with an average of 22.9 °C in 2015 and 19.8 °C in 2016.

### Dry matter accumulation, yield and fiber quality

**Figure 1:**
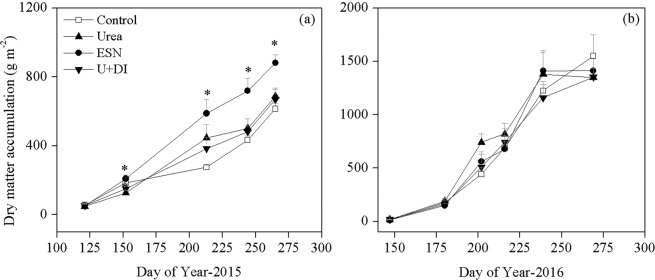
Cotton dry matter accumulation as effected by fertilizer treatments over the growing seasons in 2015 (**a**) and 2016 (**b**). Bars indicate +1 standard error of the mean, n = 4. *Indicates significant treatment effect at *P* = 0.05.

Treatment effect on dry matter accumulation varied between years (Fig. 1). In 2015, N addition generally increased the aboveground dry matter after flowering stage, with ESN had 28–43% greater dry matter accumulation than other treatments. In 2016, however, there was no treatment effect on dry matter accumulation at all sampling stages. The aboveground dry matter from the flowering to physiological maturity stage was generally greater in 2016 than 2015. At physiological maturity stage, average dry matter accumulation across treatments was 98% greater in 2016 than in 2015, being 713 and 1414 g m^−2^, respectively.

Cotton boll yield, lint percentage and lint yield were not affected by treatment (Table 1). Boll yield was 16% greater whereas lint percentage was 7% lower in 2016 than in 2015. As a result, lint yield was similar between the two crop years with an average of 1.1 Mg ha^−1^. There was no interaction effect between year and treatment on boll yield, lint percentage and lint yield.

**Table 1:** Cotton boll yield, lint percentage and lint yield as effected by fertilizer treatments in 2015 and 2016.

	Boll yield (Mg ha^−1^)	Lint percentage (%)	Lint yield (Mg ha^−1^)
**Year**			
2015	2.4 ± 0.1 b	41 ± 0.4 a	1.0 ± 0.05
2016	2.8 ± 0.1 a	38 ± 0.4 b	1.1 ± 0.06
**Treatment**			
Control	2.4 ± 0.2	40 ± 0.7	1.0 ± 0.08
Urea	2.6 ± 0.2	39 ± 0.6	1.0 ± 0.07
ESN	2.9 ± 0.2	40 ± 0.8	1.1 ± 0.06
U + DI	2.5 ± 0.2	39 ± 0.8	1.0 ± 0.09
**ANOVA** ***P*** **values**			
Year (Y)	0.045	0.000	0.302
Treatment (T)	0.345	0.291	0.426
Y × T	0.571	0.485	0.560

Values are means ±1 standard error, n = 16 for year and n = 8 for treatment.

Means within a column followed by the different letters are significantly different at P = 0.05.

All fiber quality indexes were not affected by treatment (Table 2). Some indexes, including fiber length, uniformity, strength, and spinning consistency were slightly but significantly greater in 2016 than in 2015. In contrast, the short fiber ratio and micronaire were greater in 2015 than in 2016. There was no interaction effect between year and treatment on all quality indexes.

**Table 2:** Fiber quality as effected by fertilizer treatments in 2015 and 2016.

	Length (mm)	Uniformity (%)	Strength (g tex^−1^)	Elongation (%)	Short fiber ratio (%)	Mature	Micronaire (μg inch^−1^)	Spinning consistency index
**Year**								
2015	28.8 ± 0.3 b	85.4 ± 0.3 b	28.8 ± 0.3 b	7.8 ± 0.2	3.7 ± 0.2a	0.86 ± 0.003	4.6 ± 0.1 a	135.8 ± 2.7 b
2016	30.0 ± 0.2 a	86.2 ± 0.2 a	29.9 ± 0.2 a	7.6 ± 0.1	3.3 ± 0.1b	0.85 ± 0.002	4.3 ± 0.1 b	147.7 ± 1.7 a
**Treatment**								
Control	29.2 ± 0.3	85.6 ± 0.2	29.6 ± 0.4	8.0 ± 0.2	3.4 ± 0.2	0.85 ± 0.003	4.4 ± 0.1	141.6 ± 2.3
Urea	29.0 ± 0.3	85.3 ± 0.3	28.6 ± 0.4	7.5 ± 0.3	3.9 ± 0.3	0.86 ± 0.003	4.6 ± 0.1	135.1 ± 3.0
ESN	29.5 ± 0.5	86.0 ± 0.5	29.7 ± 0.6	7.8 ± 0.1	3.3 ± 0.2	0.86 ± 0.002	4.5 ± 0.1	143.6 ± 5.7
U + DI	30.0 ± 0.4	86.3 ± 0.2	29.4 ± 0.2	7.6 ± 0.2	3.5 ± 0.1	0.85 ± 0.004	4.3 ± 0.2	146.5 ± 2.9
**ANOVA P values**								
Year (Y)	0.002	0.015	0.010	0.293	0.030	0.090	0.003	0.001
Treatment (T)	0.210	0.114	0.229	0.390	0.177	0.274	0.173	0.094
Y × T	0.749	0.572	0.240	0.889	0.408	0.890	0.879	0.770

Values are means ±1 standard error, n = 16 for year and n = 8 for treatment.

Means within a column followed by the different letters are significantly different at P = 0.05.

### Crop N accumulation, concentration and allocation

**Figure 2:**
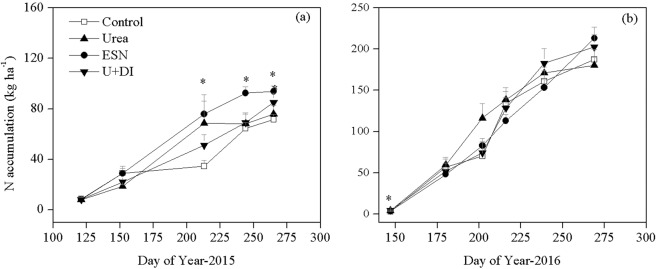
Cotton aboveground N accumulation as affected by fertilizer treatments over the growing seasons in 2015 (**a**) and 2016 (**b**). Bars indicate +1 standard error of the mean, n = 4. *Indicates significant treatment effect at *P* = 0.05.

Changes of crop N accumulation over time course followed similar trend of dry matter (Fig. 2). In 2015, fertilizer addition increased N accumulation compared to Control, starting from early flowering stage. ESN resulted in significantly greater N accumulation in aboveground than other treatments since late flowering stage and thereafter. In contrast, U + DI had comparable N accumulation as Urea over the experimental periods in 2015. In 2016, N accumulation was not affected by treatment at all stages. At physiological maturity stage, average N accumulation across treatments was 1.4 times greater in 2016 than in 2015, being 196.5 and 81.5 kg N ha^−1^, respectively.

Across the two crop years, both N concentrations and allocations in different organs at physiological maturity were not affected by treatment (Table 3). Nitrogen concentration was highest in the seed and lowest in the fiber, averaged at 27.3 and 2.4 g N kg^−1^, respectively. Nitrogen allocation followed the order of seed > straw > hull = leaf > fiber, with more than 45% of N in cotton seed. In 2016, N concentration in seed was lower whereas N in straw and leaf were higher than concentrations in 2015. As a result, less N was allocated to cotton seed whereas more N was allocated to straw in 2016 compared to 2015. Nitrogen concentrations and allocations in different organs were not affected by treatment by year interaction. The NRE was not affected by treatment, year and their interaction.

**Table 3:** Nitrogen concentrations and allocation in different organs, and N recovery efficiency (NRE, %) of cotton at physiological mature stage as affected by fertilizer treatments in 2015 and 2016.

	N concentration (g N kg^−1^)					N allocation (%)					NRE (%)
	Straw	Fiber	Hull	Leaf	Seed	Straw	Fiber	Hull	Leaf	Seed	
**Year**											
2015	8.6 ± 0.3 b	2.1 ± 0.4	10.2 ± 0.6	11.8 ± 1.0 b	30.8 ± 0.8 a	19.7 ± 1.0 b	2.5 ± 0.5	12.3 ± 1.0	13.9 ± 1.6	51.6 ± 2.4 a	5.6 ± 2.4
2016	15.8 ± 0.8 a	2.7 ± 0.1	11.8 ± 2.1	20.9 ± 0.6 a	23.8 ± 2.1 b	29.4 ± 1.6 a	2.9 ± 0.2	15.3 ± 2.4	10.9 ± 1.0	41.6 ± 2.3 b	4.8 ± 4.2
**Treatment**											
Control	11.8 ± 1.7	2.2 ± 0.2	10.5 ± 2.0	15.0 ± 2.0	27.4 ± 2.5	24.3 ± 2.7	2.5 ± 0.2	14.6 ± 3.6	12.7 ± 2.0	46.0 ± 3.6	—
Urea	12.1 ± 1.5	2.2 ± 0.2	9.4 ± 1.0	16.4 ± 2.0	28.7 ± 2.5	23.9 ± 2.1	2.5 ± 0.3	11.3 ± 2.1	12.2 ± 1.7	50.1 ± 4.1	−0.5 ± 2.3
ESN	12.0 ± 1.3	2.3 ± 0.3	12.5 ± 2.3	17.0 ± 2.2	26.8 ± 2.3	24.3 ± 1.9	2.6 ± 0.3	14.6 ± 2.7	13.4 ± 2.5	45.1 ± 3.5	10.1 ± 4.3
U + DI	13.0 ± 1.9	2.9 ± 0.7	11.7 ± 2.9	16.4 ± 2.2	26.4 ± 3.3	25.7 ± 3.5	3.2 + 0.9	14.7 ± 1.1	11.3 ± 1.6	45.1 ± 3.5	6.0 ± 4.7
**ANOVA** ***P*** **values**											
Year (Y)	0.000	0.183	0.498	0.000	0.010	0.000	0.344	0.716	0.150	0.019	0.806
Treatment (T)	0.783	0.590	0.804	0.853	0.915	0.914	0.698	0.625	0.901	0.793	0.134
Y × T	0.539	0.528	0.946	0.987	0.852	0.362	0.268	0.853	0.754	0.586	0.559

Values are means ±1 standard error, n = 16 for year and n = 8 for treatment.

Means within a column followed by the different letters are significantly different at P = 0.05.

## Discussion

Cotton is the dominant cash crop in the arid region of NW China with yield production and quality of cotton fiber being the most relevant indicators of economic benefit. This two-year field study has enabled us to evaluate the effect of EENFs on yield and quality of cotton grown under drip-fertigated system. Similar to other studies where the agronomic effectiveness of EENF products were minimal or absence^15,16^, polymer coated urea as ESN and addition of nitrification and urease inhibitors did not increase yield and fiber quality, and NRE of cotton in the current study. The absence of fertilizer source effect is highly associated with the unique conditions of drip-fertigation in the arid ecosystem.

Drip-fertigation is designed to save water consumption for crop production in arid regions where water supply is limited. In the current study, soil water content was at relatively low level which had reduced the likelihood of benefits for the coated urea and double inhibitors. Under water conservation with drip-fertigation at this study site, high soil accumulation of nitrate could have occurred due to lack of leaching in the arid ecosystem. This assumption was confirmed by a recent study of Yin *et al*., who investigated the non-growing season N_2_O emissions from the same study site and reported that the residual NO_3_^−^ concentrations in the 0–20 cm depth were high at approximately 20 mg N kg^−1^ for the unfertilized control and 30–70 mg N kg^−1^ for the N-fertilized treatments^17^. Similarly, other studies in arid regions also reported high soil residual N under drip fertigation, which could account for 35% of N applied and be readily available for crop uptake^18^.

The absence of fertilizer source effect on yield and N use of cotton could also be attributed to the relatively high application rate of 240 kg N ha^−1^, which is a typically applied rate for cotton grown in the study site but almost doubled that for the cotton production in United States^19^. Benefits of EENFs for crop production are more likely achieved at reduced N rates. For example, a nitrification inhibitor (DMPP) was not effective at standard application rate of urea but increased yields of pasture by 31% when fertilizer rate was reduced by half^20^. Li *et al*. recently summarized previous studies on EENFs in a meta-analysis and reported that EENFs were most beneficial for crop production at reduced N rates compared with conventional rates^12^. Our study highlights that it is particularly important to account for the carryover of soil NO_3_^−^ from previous years to adjust fertilizer N rates. Therefore, a fertilizer application recommendation based on soil N test is of great importance for cotton production under drip-fertigation.

As a cash crop, fiber quality must be considered to maximize the profits of growing cotton. Previous studies suggest the quality of fiber is determined by interactions between genetic, environmental and management factors^21,22^. To our knowledge, this is the first study investigating fiber quality as affected by fertilizer N management for cotton grown under drip-fertigation system. In this study, fiber quality indexes such as length, uniformity, strength, elongation, short fiber ratio, mature, micronaire, and SCI were all not impacted by fertilizer N source. Micronaire, as a measure of fiber fineness and maturity, ranged between 4.3 and 4.6 μg inch^−1^ in the current study, corresponding to the base range of 3.7 to 4.2 μg inch^−1^ according to the cotton classification in USA^23^. The SCI is a calculation for predicting the overall quality and spin ability of cotton fiber and averaged at 140 in the current study, which was greater than the values for most cotton varieties in USA^24^. These results suggest a generally high fiber quality of cotton in the study area, indicating a benefit to famer’s income from premium quality. Similar to our study, other studies also reported that cotton fiber quality was not affected by fertilizer N sources^25,26^, highlighting the challenge of improving fiber quality through management of fertilizer N. In contrast, a better fiber quality was recorded in 2016 than 2015, as suggested by higher values of fiber length, uniformity, strength and the overall SCI value. The better fiber quality in 2016 was likely obtained due to the more appropriate temperature (~22 °C) during the boll development and fibre elongation. In contrast, high temperature in 2015 during the corresponding stages (~27 °C) could have decreased the duration for boll maturation and the elongation rate of fibre. Other studies also reported that temperature is a key determinant of fibre quality through affecting fibre elongation^27,28^. These results indicate that the environmental factors are more important than fertilizer N management in determining quality of cotton.

As a polymer-coated urea, rate of N release from ESN is highly dependent on soil temperature and moisture conditions^29^. Plastic-mulch in this study could have increased soil temperature and moisture and thus enhanced N release from ESN. Zhou *et al*. reported that plastic-mulch increased soil temperature by 2.5–3.2 °C in the early stage of crop growth^30^. In this study, ESN increased dry matter and N accumulations in the cotton aboveground over the growing stages in 2015 but not in 2016, suggesting a significant influence of environment on the effectiveness of polymer-coated urea. Other study also found the efficacy of ESN was highly dependent on environment factors such as soil temperature and moisture^31^. High soil temperature for a dry ecosystem as in the current study could have stimulated the release rate of N from the polymer coat and thus shortened the duration for N availability. It was interesting to note that an increase of dry matter accumulation with ESN in 2015 did not necessarily result in an increase of yield production, highlighting the challenge to increase the fraction of dry matter allocation into seed. Still, the one-time application of ESN could save more labor cost compared with the multiple applications in the fertigation system, in spite that the price of ESN is 10–15% higher than that of urea. A systematic economic evaluation of ESN should consider yield and quality of cotton, as well as the potential benefits on environment by reducing N loss through multiple pathways such as leaching, ammonia volatilization and denitrification.

Addition of nitrification and urease inhibitors with urea did not affect dry matter, yield, as well as N accumulation, allocation and NRE of cotton in this study. In the same area as in the current study, Liu *et al*. also reported that use of a nitrification inhibitor nitrpyrin did not affect cotton yield under drip-fertigation system^14^. The ineffectiveness of inhibitors could be associated with several factors. First, plastic mulching may have increased soil temperature, resulting in a higher degradation rate of inhibitors^32^. Second, the efficacy of inhibitors may vary with soil properties such as texture, organic matter content and pH^33,34^. The coarse-texture and low organic matter content at the study site tended to reduce the adsorption of inhibitors in soil and increase the volatilization loss in arid region. A meta-analyses study revealed that nitrification inhibitors were less effective for alkaline (pH ≥ 8) and neutral (pH 6–8) soils compared to the acidic soils (pH ≤ 6)^17^. These studies, in combination with results of our study, suggest that soil properties and environmental factors should be considered when establishing the application strategies of urease and nitrification inhibitors for improving crop production. At the same site, our previous studies found use of double inhibitors with urea significantly reduced N_2_O emissions by 21%, compared with urea only^35^. Other studies also reported use of inhibitors can greatly reduce N loss pathways as nitrate leaching^6^ and ammonia volatilization^36^. The practical recommendation of enhanced efficiency fertilizers should be more focusing on the benefits to reduce N losses to environment.

## Conclusion

Through two-year field experiments, we thoroughly evaluated the impact of different fertilizer sources on yield, fiber quality and N use by cotton grown under drip-fertigated system in arid region. Results showed that there were generally no significant effects of difference N treatments on yield, fiber quality, as well as N accumulation and allocation at the physiological maturity stage. The ineffectiveness of EENFs was mostly associated with the unique conditions under the drip-fertigation in the arid ecosystem. Limited water supply could have reduced the likelihood of nitrate leaching and thus resulted in an accumulation of nitrate in surface soils, which are available for uptake by the following crops. The relatively high application rate had hindered the effectiveness of EENFs, highlighting the importance of soil N test to account for the carryover of soil nitrate to establish the reasonable N rates for crop production in the region. Overall our results highlight the challenge of adopting EENFs for the drip-fertigated cotton production system in arid northwestern China, considering the generally higher prices of EENF products than the conventional fertilizers. Further studies are needed to investigate whether the benefits of EENFs could be achieved at reduced fertilizer N rates.

## Materials and Methods

### Site description and soil properties

A field experiment was conducted at the National Grey Desert Soil Station (43°56’N, 87°28’E) of the Xinjiang Academy of Agricultural Sciences near Urumqi, Xinjiang Province, China, during 2015 and 2016 growing seasons. This region is classified as a typical continental arid climate. Annual precipitation and evaporation are 180–250 mm and 1600–2000 mm, respectively. Mean annual air temperature is 16.5 °C. The soil is classified as grey desert soil in the Chinese soil classification and Typic Argigypsids in the USDA-NRCS system and is representative of soils for cotton production in the region. In order to avoid cumulative effects of fertilizer treatments, the experiments were conducted on adjacent fields each year.

Soil (0–20 cm) was a sandy loam texture (clay 27, silt 343 and sand 630 g kg^−1^) with bulk density of 1.3 Mg m^−3^. Soil samples were collected prior to planting in each year for determination of characteristics (Table 4). Analysis of soil properties followed Carter^37^. Soil pH and EC were determined on a 1:5 soil/water suspension. Organic matter was determined by wet oxidation. Total Kjeldahl N was determined after microwave digestion with sulfuric acid and salicylic acid. Available P and K concentrations were determined by an ARL 3410 ICP unit after extraction with 0.5 M NaHCO_3_ and 1.0 M ammonium acetate, respectively. Soil texture was determined by the pipette method. Bulk density was determined by soil core method. Rainfall and air temperature data were obtained from an onsite weather station.

**Table 4:** Soil (0–20 cm) characteristic of the study site.

Year	pH	Electrical conductivity (μS cm^−1^)	Organic matter (g kg^−1^)	Total N (g kg^−1^)	Exchangeable P (mg kg^−1^)	Exchangeable K (mg kg^−1^)	Exchangeable NO_3_^−^N (mg kg^−1^)
2015	8.0	229	17.9	0.9	13.4	228	19.0
2016	8.3	120	13.7	0.8	13.0	197	8.4

### Experimental design and agronomic management

This study used a randomized complete block design consisting of an unfertilized control and application of 240 kg N ha^−1^ using (1) polymer-coated urea (ESN), (2) urea alone (Urea), or (3) urea amended with urease (NBPT) and nitrification (DCD) inhibitors (U + DI). ESN was banded in the plant row before seeding. In treatments with urea, 20% of total N was banded in the plant row before seeding, and the remaining 80% was applied by six fertigation events at 9, 11, 14, 15, 16, and 17 weeks after planting. For treatment U + DI, NBPT and DCD were applied at rate of 1% urea N, with NBPT using the same strategy as urea while DCD being all banded in the plant row before seeding. All treatments had four replicate plots. The size of each plot was 10 m × 6.4 m. In all plots, P and K fertilizers were applied at rates of 120 kg P_2_O_5_ ha^−1^ and 60 kg K_2_O ha^−1^ as Ca(H_2_PO4)_2_ and K_2_SO_4_, respectively. All P and K fertilizers were surface broadcast and then incorporated into soils before planting.

In both years, cotton (c.v. Xinluzao 48) was sown in the 4^th^ week of April under the plastic-mulch drip-fertigated system, which has been recognized as a successful practice for increasing production and water/nutrient use efficiency in the region. Details on the layout of the system was described by Ma *et al*.^35^. Briefly, four sheets of high-density and airtight transparent polythene film were mulched in each plot, separated by 50-cm bare soil between sheets. Each plastic sheet covered 4 rows of cotton with row space of 30–50–30 cm, with drip tapes being installed between the 30 cm rows. The space between plants within a row was 10 cm. In each plot, water reading meter and fertilizer tank were installed to monitor the amount of irrigation water and fertilizer N applied, respectively. Cotton was drip-irrigated for 10 times in 2015 and 9 times in 2016, with each irrigation providing about 450 mm irrigation water. Source of irrigation water was groundwater from a well at the research station. Pest and weed control followed the conventional practices in the area.

### Plant harvest and analysis

Plant samples were collected six times in each crop year for determination of dry matter and crop N accumulation, i.e. at emergence, seedling, early and late flowering, boll opening and physiological maturity stages. On each sampling occasion, two 1-m row segments were randomly selected in each plot by clipping the aboveground plant at the soil surface. Cotton crops sampled at the last occasion (physiological maturity) were divided into different organs including stem, leaf, hull, fiber and seed. All samples were oven-dried initially at 105 °C for 30 min and then to a constant weight at 80 °C for determination of plant biomass. Samples were then ground to pass a 2 mm sieve and analyzed for N concentration using Kjeldahl (8400, FOSS) digestion procedure. Concentrations were expressed on a dry weight basis and crop N accumulation was calculated as the product of concentration and dry weight. Nitrogen distribution to different organs at physiological maturity was calculated as the ratio of N accumulation in each organ to total above-ground N accumulation. Nitrogen recovery efficiency (NRE) of fertilized treatments was calculated as:

$${\rm{NRE}}=\frac{{\rm{NF}}-{\rm{NC}}}{{\rm{Applied}}\,{\rm{N}}}\times 100$$

where N_F_ and N_C_ are the above-ground N accumulation (kg N ha^−1^) of N fertilized treatments and unfertilized Control, respectively, and applied N is the applied N rate (240 kg N ha^−1^).

Cotton yield was determined by hand picking the bolls from each plot from late September to early November. Cotton boll yield (Mg ha^−1^) was determined as the dry weight of cotton bolls. Representative boll samples were ginned into separate parts of lint, seed and trash. Lint percentage, calculated as (100 × lint weight)/(weight of lint + seed + trash), was used to convert cotton boll yield to lint yield. A subsample of the lint cotton from each plot was analyzed for fiber quality index including length (mm), uniformity (%), strength (g tex^−1^), elongation (%), short fiber ratio, mature, and micronaire (μg inch^−1^), using a high-volume instrument (HVI) system. Spinning consistency index (SCI) was calculated based on a regression equation which considers the measured indexes.

$${\rm{SCI}}=-\,322.98+(2.89\times {\rm{strength}})\,\mbox{--}\,(9.32\times {\rm{micronaire}})+(43.53\times {\rm{length}})+(4.29\times {\rm{uniformity}}).$$

### Statistical analysis

A two-way analysis of variance (ANOVA) was used to determine the main and interactive effects of treatment and year on cotton boll yield, lint yield, lint percentage, fiber quality index, N accumulation and N allocation to different organs. When the main or interactive effects were significant, means were compared using least significant differences. Differences were considered significant at 0.05 level of probability. All analyses were performed using SPSS 20.0 software (IBM Corp., Armonk, NY, USA).
